# 3D directional tuning in the orofacial sensorimotor cortex during natural feeding and drinking

**DOI:** 10.1101/2024.07.02.601741

**Published:** 2024-07-04

**Authors:** Victoria B. Hosack, Fritzie I. Arce-McShane

**Affiliations:** 1 Department of Oral Health Sciences, School of Dentistry, University of Washington, Seattle, WA; 2 Division of Neuroscience, Washington National Primate Research Center, University of Washington, Seattle, WA; 3 Graduate Program in Neuroscience, University of Washington, Seattle, WA

## Abstract

Directional tongue movements are essential for vital behaviors, such as feeding and speech, to position food for chewing and swallowing safely and to position the tongue for accurate sound production. While directional tuning has been well-studied in the arm region of the sensorimotor cortex during reaching tasks, little is known about how 3D tongue direction is encoded in the orofacial region during natural behaviors. Understanding how tongue direction is represented in the brain has important implications for improving rehabilitation for people with orolingual dysfunctions. The goal of this study is to investigate how 3D direction of tongue movement is encoded in the orofacial sensorimotor cortex (OSMCx) during feeding and drinking, and how this process is affected by the loss of oral sensation. Using biplanar video-radiography to track implanted markers in the tongue of behaving non-human primates (*Macaca mulatta*), 3D positional data was recorded simultaneously with spiking activity in primary motor (MIo) and somatosensory (SIo) areas of the orofacial cortex using chronically implanted microelectrode arrays. In some sessions, tasks were preceded by bilateral nerve block injections to the sensory branches of the trigeminal nerve. Modulation to the 3D tongue direction was found in a majority of MIo but not SIo neurons during feeding, while the majority of neurons in both areas were modulated to the direction of tongue protrusion during drinking. Following sensory loss, the proportion of directionally tuned neurons decreased and shifts in the distribution of preferred direction were observed in OSMCx neurons. Overall, we show that 3D directional tuning of MIo and SIo to tongue movements varies with behavioral tasks and availability of sensory information.

## Introduction

Motor and somatosensory cortical neurons have long been known to modulate their spiking activity to the direction of arm movements during reaching tasks performed by non-human primates (Georgopoulos et al., 1988; Schwartz et al., 1988a; Prud’homme and Kalaska, 1994). Similarly, neurons in the primary motor (MIo) and primary somatosensory (SIo) areas of the orofacial sensorimotor cortex (OSMCx) have been shown to modulate their spiking activity to the direction of voluntary tongue protrusion (Murray and Sessle, 1992; Lin et al., 1994) and to semi-automatic movements, such as chewing and swallowing (Sessle et al., 2005b). However, considerably less is known about how 3D tongue direction is encoded in the orofacial region during natural feeding and drinking behaviors. Due to the tongue being inside the oral cavity and thus hidden from external view, it has proved difficult to study the neuromechanical processes underlying important behaviors such as feeding, drinking, and speech. This is significant for people with sensorimotor dysfunctions in chewing, swallowing, or speaking. Because specific directional tongue movements are essential for such behaviors, understanding how tongue direction is represented in the brain may help improve evaluation and treatment strategies.

The seminal studies on the directional tuning properties of OSMCx neurons by Sessle and colleagues employed varying locations of spouts that delivered a juice reward to elicit directional tongue protrusions without tracking tongue movements. A later study incorporated tracking of 2D tongue movements using videofluoroscopy during voluntary directional protrusions (Arce et al., 2013), but the tongue trajectories were not used to study directional tuning. In all these prior studies, primates have been trained to interact with a computer display to elicit a tongue protrusion to a specific direction on cue. There is a knowledge gap on how spiking activity in the OSMCx relates to tongue movements during natural behaviors. With the development of biplanar video-radiography (Brainerd et al., 2010), it is now possible to track these 3D tongue movements within the oral cavity at a high resolution. By simultaneous recording of tongue movements and spiking activity, we have shown recently that tongue position and shape can be accurately decoded from OSMCx during feeding (Laurence-Chasen et al., 2023).

In this study, we examined both the encoding and decoding of tongue direction in the OSMCx during untrained feeding and drinking and how this differed across multiple cortical regions. Because oral somatosensation is vital for proper tongue positioning and bolus control, and when it is impaired can cause difficulty in chewing and swallowing (Smith and Cutrer, 2011), we also investigated the role of tactile feedback in the sensorimotor control of the tongue during these behaviors.

## Methods

### Experimental setup.

**Figure 1.**
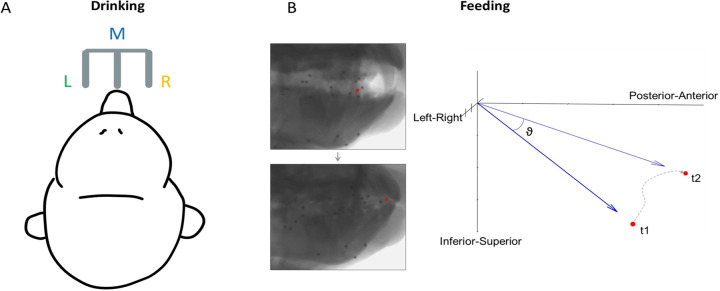
Direction of tongue motion in each behavioral task. **(A)** Schematic of the location of three spouts, left (L), middle (M), and right (R), for the drinking task. Tongue direction was categorized based on spout location. **(B)** Calculation of 3D tongue direction during feeding. ϑ is the instantaneous 3D direction of the tongue tip over a 100 ms interval between its positions at t1 and t2, where t1 = 0 and t2 = t1 + 100. The dotted line shows the actual trajectory during this interval.

Experiments were performed on two adult male rhesus macaques (*Macaca mulatta,* 9–10 kg, ages 8 and 9 years) in the University of Chicago XROMM Facility. This sample size was chosen based on precedent in the field of non-human primate motor neuroscience. All protocols were approved by the University of Chicago Animal Care and Use Committee and complied with the National Institutes of Health Guide for the Care and Use of Laboratory Animals. The subjects were seated in a standard primate chair and head-fixed to keep their head position constant during feeding and drinking trials. Each trial lasted 10 seconds. In a feeding trial, a piece of food (grape, gummy bear, pasta) of roughly the same size was presented directly to the animals’ mouth using a stylus. In a drinking trial, juice was delivered through one of three spouts positioned in front of the subject (Fig. 1A).

For some sessions, these behavioral tasks were preceded by nerve block injections (0.25% Bupivacaine HCL and Epinephrine 1:200,000, 0.25 mL/injection site) to the sensory branches of bilateral trigeminal nerves (lingual, inferior alveolar, buccal, palatine) to eliminate oral tactile sensation locally and temporarily. The nerve block was administered while the subjects were under general anesthesia, and all data were collected within 90 minutes of the nerve block. Each monkey served as its own control, with nerve block feeding data collection sessions taking place either a day before or a day after the associated control session. Nerve block drinking data collection was performed immediately following the control drinking session. Multiple datasets (40–60 trials) were collected for both subjects across multiple days. However, due to the complex and time-consuming nature of processing integrated XROMM and neural data, one session per subject, behavior, and condition was used for this study. Thus, we analyzed a total of 8 datasets.

### Video-radiography.

Prior to data collection, the animals were implanted with spherical tantalum beads (1-mm diameter) in the cranium, mandible, and the tongue, from the tip to the region of the circumvallate papillae. During feeding or drinking, the movement of these markers was recorded using high-resolution (200 Hz, <0.1 mm) biplanar video-radiography collected with Xcitex ProCapture version 1.0.3.6. The 3D positional data was obtained following the previously described X-ray Reconstruction of Moving Morphology (XROMM) workflow (Laurence-Chasen et al., 2020) incorporating the use of XMALab (Knörlein et al., 2016) and machine learning using DeepLabCut (Mathis et al., 2018) to reconstruct the kinematic data. The *x*, *y*, *z* values of the markers were then smoothed with a 30 Hz low-pass Butterworth filter and transformed into a cranial coordinate space with the origin fixed at the posterior nasal spine. Gape cycles within each feeding sequence were manually identified and categorized by cycle type (manipulation, stage 1 transport, chew, stage 2 transport, or swallow).

### Electrophysiology.

Under general anesthesia, a microelectrode array was chronically implanted in four areas of the left hemisphere (Supplementary file 1, Fig. 1): rostral MIo (96-electrode Utah array; electrode length: 1.5 mm), caudal MIo (32-electrode Floating microelectrode array (FMA), electrode length: 3.0–4.5 mm), area 1/2 (96-electrode Utah array, electrode length: 1.0 mm), and area 3a/3b (32-electrode FMA, electrode length: 4.0–8.7 mm). The neural data was recorded using Grapevine Neural Interface Processor (Ripple Neuro, Salt Lake City, UT). Signals were amplified and bandpass filtered between 0.1 Hz and 7.5 kHz and recorded digitally (16-bit) at 30 kHz per channel. Only waveforms (1.7 ms in duration; 48 sample time points per waveform) that crossed a threshold were stored and offline spike sorted (Offline Sorter, Plexon, Dallas, TX) to remove noise and to isolate individual neurons. The channel name assigned to each recorded neuron was kept consistent between control and nerve block data for comparison.

### Data analysis.

3D tongue kinematics were recorded simultaneously with the neural data in all behavioral sessions. All data analyses were performed in MATLAB 2022b (MathWorks, Natick, MA). For feeding, the instantaneous 3D direction of the tongue tip marker for every 100 ms throughout each gape cycle was calculated as:

(1) $$3D\;\;angle,\vartheta =\tan^{-1}\left(\frac{\left\|v_{1}\times v_{2}\right\|}{v_{1}\cdot v_{2}}\right)$$

Where $v_{1}$ is the *x*, *y*, *z* position at the start of each 100-ms interval and $v_{2}$ is the position at the end (Fig. 1B). These directions were then categorized based on whether the movement was negative or positive relative to the horizontal plane (Left/Right), the sagittal plane (Inferior/Superior), and the *x* axis (Posterior/Anterior). This resulted in eight directions: AntSupL, AntSupR, AntInfL, AntInfR, PostSupL, PostSupR, PostInfL, and PostInfR. An equal number of 100-ms intervals from each of these directions was sampled, and spike data during each was used for neural analysis. For comparison with the drinking task, the sign was determined relative to the horizontal plane, with rightward tongue movement being positive. This is also the plane of motion which has been the least studied. These left-right directions were categorized into six 10°-bins with a total range of −30° to 30°, which encapsulated the majority of the observed distribution of directions in each subject. Lingual yaw (transverse rotation) and pitch (elevation/depression) were also calculated to compare tuning across the lateral and vertical components of tongue direction (Supplementary file 2, Equation 2). For drinking, the direction was determined by which of the three spouts juice was dispensed from during each lick. The spiking activity used for neural analysis of the drinking task was from intervals of ±250 ms around each minimum protrusion of the tongue. As 100 ms was not sufficient to capture the full range of tongue motion during each drinking cycle, the length of time used was increased to allow a clear distinction between the three directions.

Kinematic performance for feeding was determined by the spread of tongue directions observed across trials. For drinking trials, performance was determined by the variance of endpoint positions as well as by the proportion of “failed” cycles, where the monkey missed the correct spout location with their tongue tip. The difference between control and nerve block performance was evaluated using a two-tailed t-test and f-test.

Tongue directions were subsequently compared with the firing rates of individual neurons across cortical areas. Neurons were identified as directionally modulated if their mean firing rate varied significantly across directions (Kruskal-Wallis, *p* < 0.05). The proportions of neurons that were found to be directionally tuned were compared across groups using a chi-square test. Then, multiple linear regression was used to determine if the firing of each neuron fit the cosine tuning function that has been previously described for the arm area of the motor cortex (Schwartz et al., 1988b). To accomplish this the directional components of a unit vector representing each group of directions were calculated. For neurons that fit the tuning function, a preferred direction (PD) in 3D space was estimated. These PDs are distributed around a unit sphere, with the origin representing the start of the movement. The directional index was calculated as a measure of the depth of directional tuning. The PD for the drinking task was determined as the direction for which a neuron exhibited its maximal firing rate. Similarly, a PD across the left-right feeding directions was determined for comparison. Circular concentration (k-test) to compare distributions of PDs during feeding and polar plot generation (Fig. 6A and Fig. 10A) were performed using the CircStat MATLAB toolbox (Berens, 2009). For drinking, distributions of PDs were compared using a chi-square test.

### Decoding tongue direction.

The ability to predict tongue direction from spiking activity of MIo and SIo neurons was evaluated using a K-nearest neighbor (KNN) classifier. The Euclidean distance was used to identify nearest neighbors, and the number of nearest neighbors used was K = 7. This K value was determined after testing different Ks which yielded comparable results. The feature was the firing rate of each neuron over each trial: every 100 ms throughout feeding sequence, or 100 ms centered at minimum tongue protrusion during drinking. As a more direct comparison to the drinking, feeding directions were combined into three groups representing left, middle, and right movement directions. The decoder was trained on 80% of trials and tested on the remaining 20%, then decoder performance was determined by the percentage of test trials where the direction of movement was correctly decoded from the neural data. We ran 100 iterations of the classifier using a different set of randomly selected training and test trials then calculated the average performance. The same sets of training and test trials were used for decoding from simultaneously recorded MIo and SIo data. However, our recorded populations were of variable sizes, and decoding performance was found to be related to the number of neurons in the ensemble (Supplementary file 1, Fig. 6). Because the smallest population of neurons we recorded was 28, we selected 28 random neurons from the larger populations for each iteration. Based on the positive relationship between population size and decoding accuracy, we expect that performance would increase with more neurons. These results will show whether tongue direction can be decoded from a very small number of neurons. We fit a linear regression model with interactions to compare decoding performance across the other variables in the experiment.

## Results

Past studies have investigated directional tuning of OSMCx neurons during trained tongue-protrusion tasks. Here we study two untrained and natural behaviors, feeding and licking (i.e., “drinking”) from a spout. In the feeding task, there is no guidance on how to move the tongue, whereas in the drinking task, the direction of movement is guided by the location of the spout. By comparing how modulation to tongue direction differs between natural tasks with and without specific directional input, we can examine how the movement of the tongue is coordinated by the OSMCx under different behavioral contexts.

In both tasks, many neurons exhibited significant modulation to tongue direction (Kruskal-Wallis, *p* < 0.05), though there were diverse patterns of spiking activity. Figure 3A illustrates the directional tuning for a neuron in MIo during natural feeding. In this example the neuron is strongly tuned to posterior-anterior and inferior-superior movement directions but shows no strong preference across the left-right axis. Many of the recorded neurons in each population behaved in a similar fashion, with peaks most frequently observed toward the anterior and superior directions.

In the feeding task, over 80% of neurons in the motor areas (rostral and caudal MIo) exhibited tuning to 3D direction (Fig. 3B, left), compared to only over 40% of neurons in the somatosensory areas (areas 3a/3b, 1/2). The difference in the proportion of MIo and SIo neurons exhibiting directional tuning during feeding was significant in both subjects (Chi-square, *p* < 0.001). Fewer neurons were tuned during swallows than chews (Supplementary file 1, Fig. 4; MIo: *p* < 0.05, SIo: *p* > 0.1). In the drinking task, greater than 50% of neurons across all recorded areas in both subjects exhibited tuning to the direction of tongue protrusion (Fig. 3B, right). Unlike the feeding task, the difference between the proportion of directionally tuned neurons in MIo and SIo was not significant in the drinking task for either subject (Chi-square, *p* > 0.1). We then used multiple linear regression to determine whether the firing of these neurons varied in an orderly fashion with tongue direction. Of the neurons that were found to be directionally modulated during feeding, 86.36% in MIo and 75.32% in SIo also fit the tuning function (*F*-test, *p* < 0.05). The distribution of preferred directions was non-uniform across the unit sphere (Fig. 4A; Rayleigh test, *p* < 0.001), with the highest density of PDs toward the most inferior and superior directions. The distribution of the directional index was skewed with a mean of 0.5374 (Fig. 4B).

We also analyzed the tuning of these neurons to the lateral (yaw) and vertical (pitch) components of tongue direction during feeding. Figure 5A shows peak activity of a neuron in MIo and in SIo at varying degrees of pitch and yaw. Overall, there was a higher proportion of neurons tuned to pitch than yaw (Fig. 5B). More neurons in MIo than in SIo exhibited tuning to both yaw and pitch (Chi-square, yaw: *p* < 0.08, pitch: *p* < 0.001), consistent with our 3D direction results. Of those neurons that were directionally tuned, we generally observed sharper and more narrow tuning curves in MIo, compared to the broader tuning of SIo (Fig. 5A). More information about directional tuning to yaw and pitch is included in Supplementary file 2. Due to the similarities between the results from each of the three angles, we primarily discuss the 3D angle during feeding in this paper, but these additional measures serve to inform our analysis of directional tuning in terms of its horizontal and vertical components.

For all subsequent analyses, we combined neurons in rM1 and cM1 as MIo and area 1/2, 3a/3b as SIo because of the small number of recorded neurons in some cortical regions (Supplementary file 1, Table 1). We evaluated whether the distribution of PDs across left to right directions differed between tasks and across cortical regions. Intrinsic and extrinsic tongue muscles are involved in shaping the tongue (e.g., elongation, broadening) and positioning the tongue (e.g., protrusion/retraction, elevation/depression), respectively. These muscles receive bilateral motor innervation except for genioglossus. The straight tongue protrusion requires the balanced action of the right and left genioglossi while the lateral tongue protrusion requires the action of the contralateral genioglossus. Since genioglossus receive unilateral innervation, we expected to see more neurons in the left MIo/SIo to have PDs for tongue movements that were leftward protrusion through the activation of the right genioglossus. Indeed, the PD distributions of directionally tuned neurons during feeding had peaks towards tongue movements to the left, except for SIo of Monkey R (Fig. 6A). Similar results were found with the distributions of preferred yaw during feeding (Supplementary file 2, Fig. 4). While the PD distribution in feeding were comparable between MIo and SIo in both subjects (circular k-test, *p* > 0.1), there was a significant difference during drinking between the PD distributions of MIo and SIo neurons in Monkey R (Chi-square, *p* < 0.001), but not in Monkey Y (*p* > 0.09). As in feeding, a majority of MIo and SIo neurons in Monkey Y had left protrusion as their PD. In contrast, proportion of MIo neurons with PDs for left and right protrusions were comparable in Monkey R, which might suggest involvement of muscles other than the right genioglossus during drinking.

### Effects of nerve block

**Figure 2.**
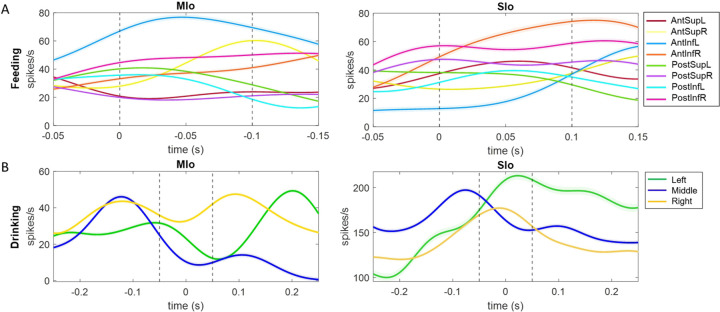
Examples of single neuron activity in relation to tongue direction. **(A)** Each peri-event time histogram (PETH and ±1 SE, smoothed by a 50-ms Gaussian kernel) corresponds to spiking activity during a 200 ms window for a specific range of tongue direction for feeding trials. Dashed lines indicate the 100-ms interval used for calculating the tongue direction. **(B)** PETHs for drinking trials with the same spout, centered at the point of minimum protrusion of the tongue (0 s).

**Figure 3.**
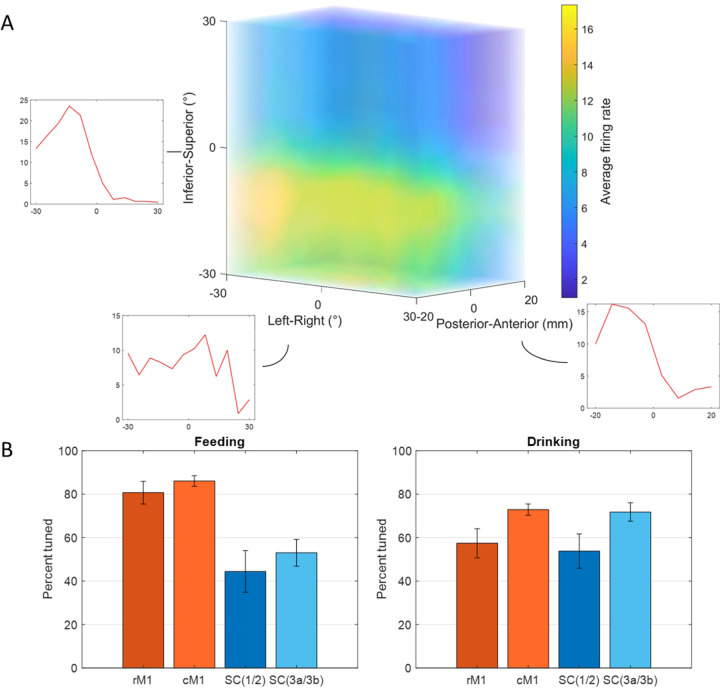
Directional tuning of neurons during control tasks. **(A)** 3D firing rate map of a neuron in MIo during feeding. Smaller inset plots are 1D tuning curves across each axis. **(B)** Percentage of neurons tuned to direction, combined for both subjects. Recordings were taken from four areas of the OSMCx: rM1 - rostral M1, cM1 - caudal M1, SC(1/2) - area 1/2, and SC(3a/3b) - area 3a/3b. Error bars represent ±1 SE.

Sensation plays a key role in tongue positioning and movements for natural behaviors. During ingestion, tactile feedback is necessary for locating the bolus, preventing tongue bites, feeling where the drinking spout is, and identifying when it is safe to swallow. To evaluate the role of oral sensation, we used a bilateral oral nerve block to temporarily eliminate tactile sensation in the oral cavity and observe how the control of tongue movement was impacted. Below we show how the loss of sensation affected both tongue kinematics and directional tuning of neurons during feeding and drinking. To verify that differences between the control and nerve block conditions were due to the loss of sensory feedback and not as a result of other factors such as sedation and injection, a sham experiment was conducted where saline was administered to the injection sites instead of nerve block. No significant changes to tongue kinematics were observed following the sham experiments (Supplementary file 1, Figs. 2 and 3).

#### Tongue kinematics.

In feeding, the mean and overall spread of directions were significantly different between the control and nerve block conditions (t-test, *p* < 0.01 and f-test, *p* < 0.001). There was a shift towards a smaller range of 3D directions in Monkey R, whereas there was a shift towards a broader distribution in Monkey Y under the nerve block condition (Fig. 7A). The positions of maximum protrusion of the tongue during drinking, i.e., the endpoints, were also affected by the loss of sensation. These endpoints represent the planned target position of the tongue to receive the juice reward from a specific spout. In the control drinking task, the endpoints for each spout location were very distinct. In contrast, the endpoints of tongue movements in nerve block exhibited a greater overlap across locations and more variance in all three axes of motion, i.e., Posterior-Anterior, Inferior-Superior, and Left-Right (Fig. 7B).

Compared to the control, the trajectories of the tongue tip in the nerve block condition during drinking had a smaller range of Left-Right values. Visually the tongue trajectories toward the different spout locations were messier and less distinct from each other as was observed in failed cycles where the tongue tip missed the location of the correct spout (Fig. 8). Failed cycles are those whose endpoint positions are greater than ±2 standard deviations from the mean endpoint position. In both monkeys there was a significant increase in the average distance from the mean endpoint position, though this difference was much greater in Monkey R (Fig. 7C). We noted a difference between subjects in the frequency of failed cycles and the range of left-right tongue movements under nerve block. This may reflect a possible compensatory strategy of reaching the drinking spouts with an adjacent region of the tongue, instead of contacting the right or left spout with the ipsilateral tongue in Monkey R.

#### Directional tuning of MIo and SIo neurons.

Loss of oral sensation also affected the proportion of directionally tuned neurons and the overall distribution of PDs, though the pattern of changes differed between subjects. Following nerve block, MIo and SIo showed overall decreases in the proportion of directionally modulated neurons in both tasks (Fig. 9A; Chi-square, MIo: *p* < 0.001, SIo: *p* < 0.01). We then verified whether these changes could be attributed to neurons gaining or losing directional tuning in nerve block. The majority of neurons in MIo and SIo for both tasks remained directionally tuned, and the proportions of neurons that gained or lost directional tuning were similar between cortical regions and subjects (Fig. 9B).

The proportion of neurons that lost and gained directionality were similar for MIo and SIo during feeding (Chi-square, *p* > 0.1). During drinking, more neurons lost directional tuning in SIo than in MIo in Monkey R (*p* < 0.01), but for Monkey Y the proportion of neurons that gained directional tuning was higher in SIo than MIo (*p* < 0.05). When comparing between behaviors, there were similar proportions of neurons that gained and lost directional tuning for Monkey R (Chi-square, *p* > 0.1), but for Monkey Y there was a higher percentage of neurons that gained directionality in both MIo and SIo during drinking than during feeding (*p* < 0.05).

As for PD distribution, we found a significant shift in the mean PD in the nerve block condition of the feeding task; the mean PD of MIo neurons in both subjects shifted clockwise toward the center (0°) and exhibited a more uniform distribution (Fig. 10A; circular k-test, *p* < 0.01). The PD shift for the population of neurons in SIo was inconsistent, with only Monkey R showing a significant counterclockwise shift (*p* < 0.05). In drinking under nerve block, the PD distribution in MIo shifted from right to left in Monkey R and from left to right in Monkey Y (Fig. 10B; Chi-square, Y: *p* = 0.04), whereas the PD distribution of SIo neurons shifted towards the right in both animals (Chi-square, R: *p* = 0.02).

### Population decoding of tongue direction

Next, we evaluated the directional information contained in the population activity. We employed a k-nearest neighbor (KNN) to predict the tongue direction from the firing rates of each neuron over a 100 ms interval. The KNN classifier was able to decode the direction of tongue movements above chance level for both feeding and drinking behaviors. Due to the large variability in the population size of MIo and SIo neurons which could affect the results, we compared decoding accuracies by downsampling the number of neurons used for decoding to that of the smallest recorded population, N = 28 (Fig. 11). Through multiple linear regression, the effects of the behavior (feeding/drinking), region (MIo/SIo), condition (control/nerve block), and interactions between these factors on decoding accuracy were evaluated. The performance of the decoder was on average 20% higher in the drinking task than in the feeding task. Decoding accuracy was greater using MIo than SIo by 13%, and in the absence of tactile sensation there was no significant decrease in the performance of the KNN classifier. Between subjects, decoding accuracy was similar in the feeding task but 17% higher for Monkey R than Monkey Y in the drinking task, indicating a higher inter-subject variability in directional information for the drinking task.

## Discussion

The aim of this study was to determine how 3D directional information is represented by MIo and SIo neurons during natural feeding and drinking behaviors, and how it is affected by the loss of tactile sensation. We found high proportions of directionally tuned neurons and above chance level decoding of directions from neuronal activity. When comparing control sessions to sessions preceded by the bilateral nerve block, we found significant changes in the direction of tongue movement, the proportion of directionally tuned neurons, and the distribution of the PDs of OSMCx neurons.

### Directional tuning in the oral sensorimotor cortex

This is the first study to investigate directional tuning of OSMCx neurons to 3D tongue direction continuously over time during macaque feeding and drinking. Unlike previous similar studies, the monkeys were not trained to reach specific targets and were instead allowed to eat and drink relatively naturally. In prior work, it was found that the OSMCx encodes direction similarly to the arm region during a trained tongue protrusion task (Murray and Sessle, 1992; Sessle et al., 2005a; Arce et al., 2013). In the present study, results from the more natural drinking were consistent with findings in the tongue protrusion task. We found similarly high proportions of directionally modulated neurons, and the PD distributions in the control condition were similar to those of the trained tongue protrusion. More importantly, by comparing the two natural behaviors, we found that the directional information in OSMcx was lower in feeding than in our drinking task, indicating a higher cortical engagement in behaviors characterized by a greater degree of volition.

Interestingly, the proportion of directionally tuned neurons in the feeding task exhibited a significant disparity between MIo and SIo, suggesting a higher directional information in MIo for tongue movements in feeding. The difference between the directional tuning of neurons in feeding compared to drinking suggests that directional tuning properties of neurons is modifiable by the behavioral task. In the drinking task, the tongue moves to discrete locations to receive the juice reward, thus there is greater involvement of SIo in determining tongue direction and reliance on sensory feedback. In contrast, tongue movements during feeding may have minimal intention or volition in moving the tongue in a specific direction. We considered that the difference in the directional tuning of SIo neurons between the two behaviors could be due to the different time intervals used for each task since the period around minimum tongue protrusion in the drinking may contain more of the sensory inputs from the previous lick. However, when sampling spiking activity from an earlier time period in feeding, the percentage of directionally tuned SIo neurons was still significantly lower than MIo (Chi-square, *p* < 0.001, data not shown). Shifting the time period of the drinking trials to be after minimum protrusion also did not lead to a significant difference between MIo and SIo (*p* > 0.1). Our results suggest that the somatosensory cortex may be less involved than the motor areas during feeding, possibly because it is a more ingrained and stereotyped behavior as opposed to tongue protrusion or drinking tasks, where the subject must specifically reach out with the tongue to feel the spout and juice reward. It has been found that proprioceptive feedback is suppressed by spinal interneurons during locomotion, another semi-automatic behavior, to promote smooth and rhythmic movements (Koch et al., 2017; Dallmann et al., 2023). A similar mechanism may exist for feeding, where unnecessary or confounding sensory information about tongue movement is more inhibited during chewing or swallowing.

**Figure 4.**
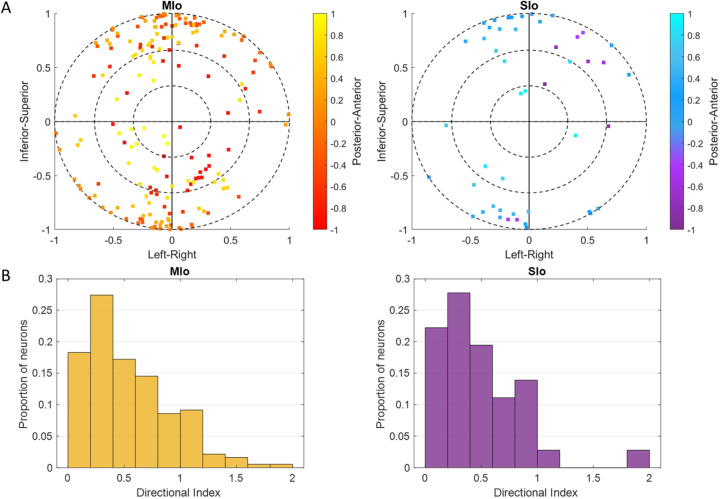
Cosine tuning of MIo and SIo neurons. **(A)** Distribution of 3D preferred directions in unit sphere for neurons that fit the tuning function during feeding, combined for both subjects. The origin represents the start of a movement. Color bar represents posterior-anterior axis. **(B)** Distribution of the index for the depth of directional tuning, combined for both subjects.

**Figure 5.**
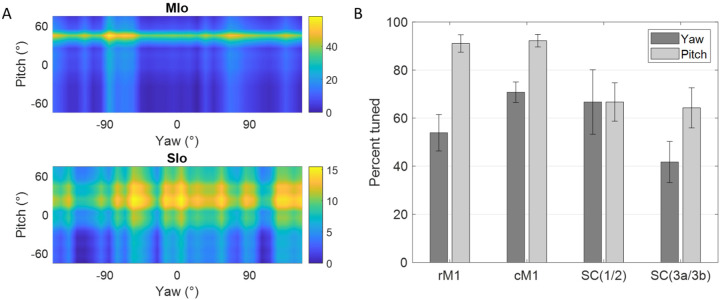
Directional tuning to yaw and pitch during feeding. **(A)** Firing rate maps of a neuron in MIo and in SIo across yaw and pitch angles. Firing rates were averaged across all 100 ms feeding intervals within a 10° range. **(B)** Proportion of neurons tuned to yaw and pitch, combined for both subjects. Recordings were taken from four areas of the OSMCx: rM1 - rostral M1, cM1 - caudal M1, SC(1/2) - area 1/2, and SC(3a/3b) - area 3a/3b. Error bars represent ±1 SE.

**Figure 6.**
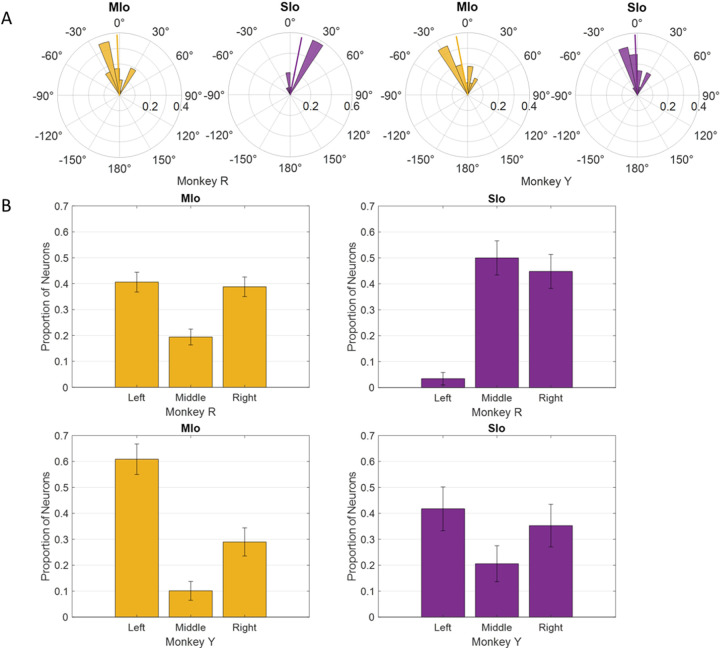
Distribution of PDs in MIo (yellow) and SIo (purple) neurons during control feeding **(A)** and drinking **(B)**. For the feeding task, polar plots are split into 10° bins with thick colored lines representing the mean PD. For the drinking task, error bars represent ±1 SE.

**Figure 7.**
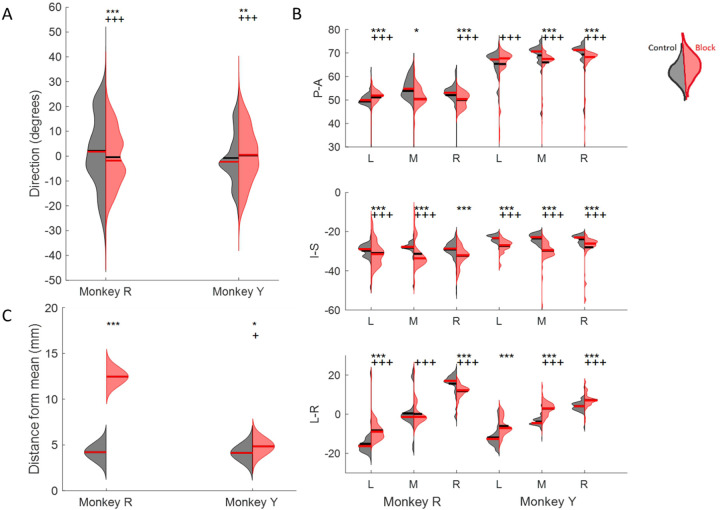
Effect of nerve block on direction of tongue movement. **(A)** Distribution of tongue directions during feeding. **(B)** Variance in 3D trajectory endpoints during drinking (Posterior-Anterior, Inferior-Superior, Left-Right) for each direction: left (L), middle (M), right (R). **(C)** Variation in the distance of drinking endpoint positions from the mean endpoint. Left halves of hemi-violins (black) are control and right halves (red) are nerve block for an individual. Horizontal black lines represent the mean and horizontal red lines the median. Results of two-tailed t-test and f-test are indicated by asterisks and crosses, respectively: *,† p < 0.05; **,†† p < 0.01; ***,††† p < 0.001.

**Figure 8.**
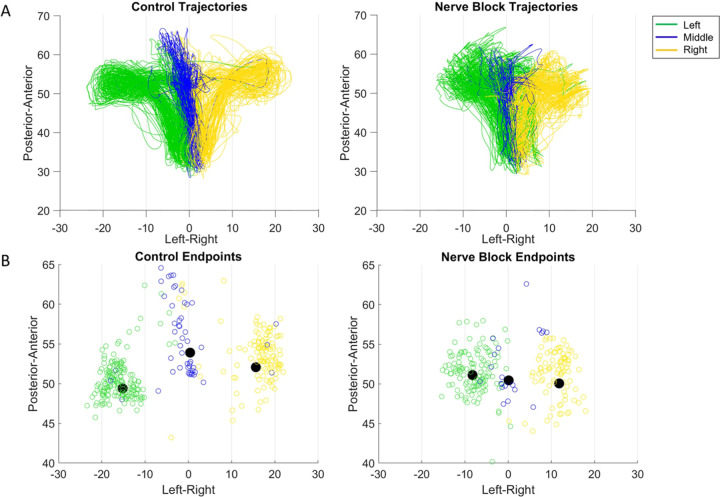
Effect of nerve block on drinking kinematics in Monkey R. **(A)** Tongue tip trajectories from starting position to one of three drinking spouts in the control and nerve block conditions. **(B)** Drinking trajectory endpoints, where the black dot represents the mean endpoint position.

**Figure 9.**
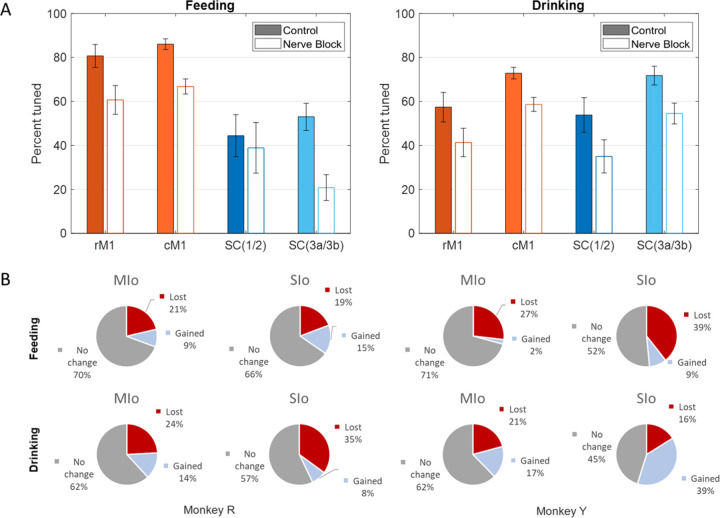
Effects of nerve block on directional tuning of OSMCx neurons during feeding and drinking tasks. **(A)** Percentage of directionally tuned neurons in four areas: rM1 - rostral M1, cM1 - caudal M1, SC(1/2) - area 1/2, and SC(3a/3b) - area 3a/3b. Filled in bars represent control while empty bars represent nerve block. Error bars represent ±1 SE. **(B)** Percentage of MIo and SIo neurons which gained or lost directionality with the addition of nerve block.

**Figure 10.**
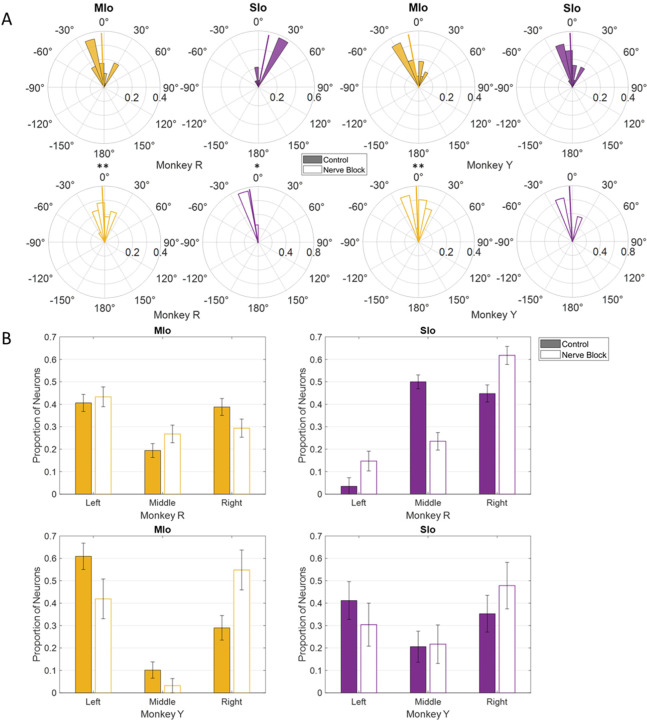
Effects of nerve block on the distribution of PDs of MIo (yellow) and SIo (purple) neurons. **(A)** For the feeding task, polar plots are split into 10° bins with thick colored lines representing the mean PD. Significant circular concentration test (k-test) comparing control and nerve block are indicated by asterisks: *p < 0.05; **p < 0.01; ***p < 0.001. **(B)** For the drinking task, error bars represent ±1 SE. Filled in bars represent control while empty bars represent nerve block.

**Figure 11.**
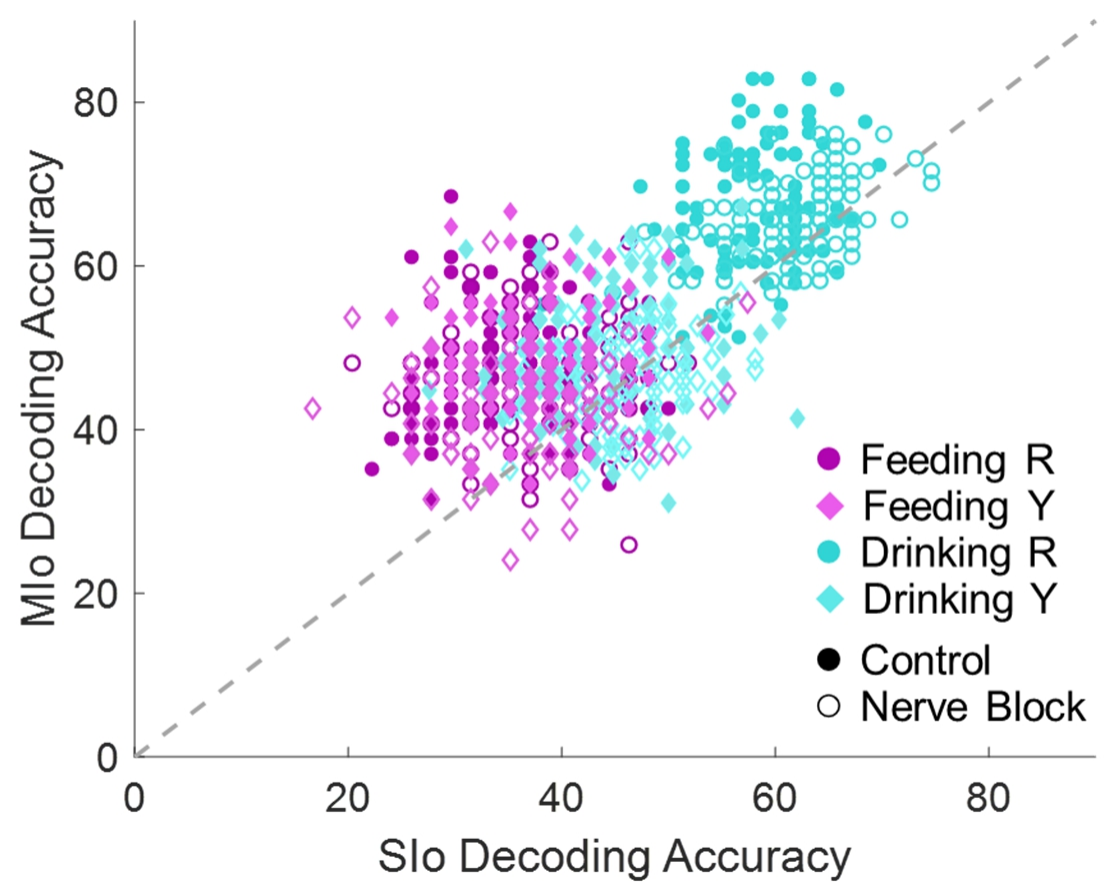
Decoding accuracies from neuronal populations of equal size (N=28). Data shown separately for each subject, behavioral task, and condition. The dashed line signifies equal decoding performance for MIo and SIo. Chance level is 33.33%. Decoding accuracies from full populations are included in Supplementary file 1, Fig. 5.

We additionally found different distributions of PDs. This signifies that there are more neurons in the left hemisphere contributing toward one direction of tongue movement, suggesting that there is some laterality in the PDs of OSMCx neurons that varies between individuals. The preferred chew side was the same for both monkeys (i.e., right side), so this finding may be somewhat contrary to previous results in humans examined using fMRI showing that hemispheric differences in sensorimotor activity during voluntary tongue movements are related to the preferred chew side (Shinagawa et al., 2003). It is possible that the difference between the two subjects is related to the difference in recording locations, with Monkey Y’s being more lateral and therefore closer to the swallow area of the cortex than Monkey R’s (Supplementary file 1, Fig. 1). Monkey Y had a higher proportion of neurons that were tuned to tongue direction during feeding compared to Monkey R (Supplementary file 1, Fig. 4), but fewer during drinking. An avenue for further study could be a unilateral nerve block on the preferred side to measure how the unaffected side of the tongue compensates for the lack of sensation in the affected side. A previous study found that unilateral lingual nerve transection in pigs alters the coordination of the ipsilateral tongue side during chewing (Montuelle et al., 2020). The tongue is a complex group of muscles, with intrinsic muscles primarily contributing to the shape and size of the tongue and extrinsic muscles contributing more to the positioning of the tongue. Therefore, it is possible that the neurons which are strongly tuned to tongue direction have direct connections to the extrinsic muscles on the ipsilateral side. Looking at how each side of the tongue responds independently to unilateral nerve block, and how this interacts with directional preference may give us more information about how the unique structure of the tongue is coordinated.

### Effects of the loss of tactile feedback

It was previously found that the administration of bilateral nerve block impaired feeding performance and tongue jaw coordination, and that it slowed the feeding sequence (Laurence-Chasen et al., 2022). The present study shows that directional movement of the tongue (*kinematics*) and the spiking activity of MIo and SIo were also affected. In the absence of sensory feedback, there were decreased proportions of directionally tuned neurons, shifts in the distributions of PDs, less distinct groups of drinking endpoints, more failed drinking cycles, and shifts in the distribution of tongue directions across feeding cycles. The reduction in directionally tuned neurons in feeding may be attributed to a more cautious approach to avoid injury in the absence of sensory feedback. This may be indicative of a reversion to previously known feeding patterns, causing the monkey to be less likely to adjust the feeding sequence when unable to feel the location of the bolus.

Our results demonstrate the importance of tactile feedback in directing tongue movement especially during drinking. Tactile feedback allows for adjustments of the direction of tongue movement to achieve contact with the spout within one or two licks. Without this information, the animal is only guided by whether the juice reward was received or not. In this case the subjects may utilize gustatory feedback to aid in locating the correct spout. In a recent optogenetic inhibition study on licking in mice, it was found that the tongue/jaw regions of the somatosensory cortex were necessary for proper tongue targeting but not for the core motor capabilities of the tongue (Xu et al., 2022). The decrease in the range of tongue motion that we observed is therefore most likely due solely to the loss of sensory feedback, not a loss of motor function itself. It is also worth noting that our drinking setup does not completely eliminate visual feedback for the monkey, which could also contribute to readjusting tongue position. However, oral sensory loss alone had a significant effect on the monkeys’ performance in this task, and tongue movements are not typically guided by visual inputs as the tongue is usually not visible within the oral cavity.

There were varying effects of the nerve block on directional tuning. The proportion of directionally tuned MIo neurons increased in Monkey R but decreased in Monkey Y during feeding, while the proportion of directionally tuned SIo neurons decreased in Monkey R but increased in Monkey Y during drinking. An increased proportion of neurons were tuned to pitch in Monkey Y with nerve block, whereas there was a decrease in Monkey R. There was no significant shift in the distribution of PDs in only the SIo of Monkey Y during both feeding and drinking with nerve block. Monkey Y also did not exhibit the same increase in failed cycles that was observed in Monkey R and did not display as significant a shift in the proportion of neurons that were directionally tuned during drinking as Monkey R did. Interestingly, there was a relatively large proportion (40%) of SIo neurons in Monkey Y that gained directional tuning following sensory loss compared to Monkey R (8%) during drinking. The loss of sensation affected these properties to a lesser extent in Monkey Y, possibly due to differences in the array locations or differing compensatory strategies between the two subjects. Due to the high variability between individuals in their response to nerve block, studying additional subjects would expand our understanding of how the OSMCx adapts to coordinate tongue movement following sensory disruption.

A limitation of the present study is that head position and hand movement were restrained, especially since there may be significant interaction between hand and orofacial regions while handling food or drink. The hand and orofacial areas are next to each other in the cortex and highly interconnected (Forrester and Rodriguez, 2015), and researchers have found an area in mice that coordinates hand-to-mouth movements during natural feeding (An et al., 2022). Fully natural feeding would involve holding food up to the mouth, as well as free head movement, which would make tracking of the marker positions difficult under this experimental setup. Further improvements in our ability to track tongue movements would be necessary to study more complex feeding sequences.

### Population decoding of tongue direction

The ability of a population of neurons to decode tongue direction from spiking activity over a short time interval reveals the extent to which the OSMCx is informed of tongue direction during natural behaviors, and thus, it has implications for the control of neuroprostheses. The direction of voluntary tongue protrusion was able to be decoded from simultaneously recorded MIo and SIo populations (Arce et al., 2013). Here, we showed that this could be applied to a cyclic, naturalistic behavior, and that instantaneous 3D tongue direction could be decoded. We observed an increase of up to 10% in decoder performance with the full populations of recorded neurons compared to the results from only 28 neurons, so we expect that decoding accuracy would increase to a similar extent with a much larger population (>100 neurons). Although downsampling leads to an overall decrease in decoding accuracy, it allows for the comparison across other variables. The higher decoding accuracy in drinking than in feeding suggests that directional information in OSMCx neurons is higher in drinking from varying spout location. As expected, decoding from MIo yielded higher accuracy than SIo in both behaviors, though the distinction was greater during feeding, consistent with our directional tuning results. These results support the well-established role of MIo in the control of movement parameters, especially direction.

The changes to decoding accuracy with nerve block were less significant than the other changes we observed. There was a slight decrease in decoding accuracy with nerve block in MIo and no difference overall in SIo, indicating that MIo and SIo still contain substantial information about tongue direction even without tactile feedback. While it was expected that MIo may still be informed about the movement of the tongue, it is surprising that performance using SIo remained consistent and even increased with nerve block. This may be evidence that another area is supplying SIo with information to compensate for the lack of tactile feedback, such as information from the taste buds.

### Clinical implications

This study offers new information about the important role of sensorimotor integration in controlling tongue direction during natural behaviors. There is a high degree of directional information contained in the spiking activity of the orofacial cortex, especially in the motor areas. The effect of the bilateral nerve block serves to enhance our understanding of the processes affected by oral sensorimotor dysfunctions such as trigeminal neuropathies. It demonstrates the importance of oral sensation for supporting the full range of directional motion, but also shows that significant directional information can be extracted even in the absence of tactile feedback.

This type of knowledge can inform the diagnosis and rehabilitation of orolingual dysfunctions. There have also been advancements in brain-computer interface (BCI) by decoding the real-time signals of arm region of the motor cortex to control prosthetic arm movement (Collinger et al., 2013; Vilela and Hochberg, 2020) or muscle stimulation (Ethier and Miller, 2015), as well as efforts to restore sensory feedback by stimulating correct areas of somatosensory cortex in response to sensors on a prosthetic (Tabot et al., 2013; Flesher et al., 2021). Therefore, information about how a different feature of movement is encoded in the OSMCx may prove helpful in the development of therapies or soft prosthetics for people with impaired orolingual function or glossectomy.

## Supplementary Material

Supplement 1

Supplement 2
