# Supplementary material for: 3D directional tuning in the orofacial sensorimotor cortex during natural feeding and drinking
Source: bioRxiv. 2025 Apr 24:2024.07.02.601741. Originally published 2024 Jul 4. Preprint. [Version 3] doi: 10.1101/2024.07.02.601741 (PMC11244964; doi:10.1101/2024.07.02.601741)
Supplement: Supplement 1 [file media-1.pdf]

Supplementary file 1

Supplementary figures and tables

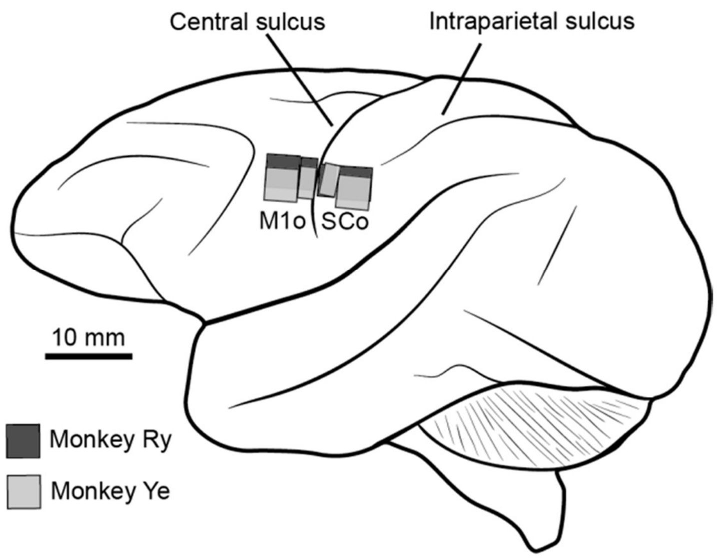

Supplementary file 1 - Figure 1. *Microelectrode array locations. Squares represent Utah arrays and rectangles represent floating microelectrode arrays. Drawing is to scale and array locations were taken from surgical photographs. Adapted from Supplementary Figure 8 of "Robust cortical encoding of 3D tongue shape during feeding in macaques" (Laurence-Chasen et al., 2023).*

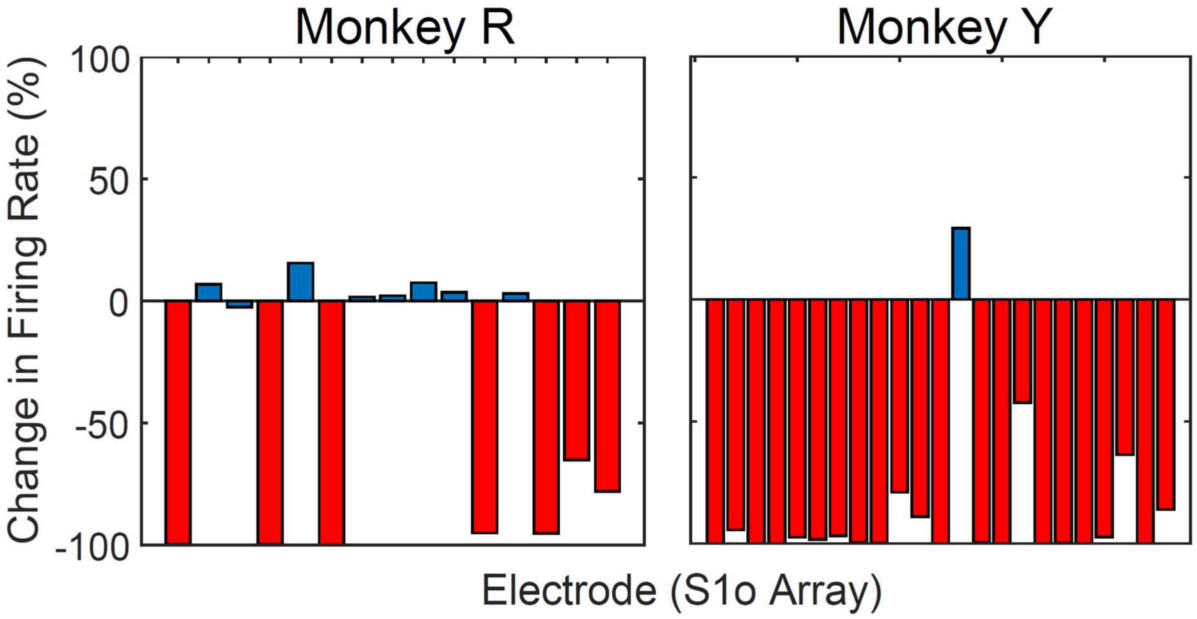

Supplementary file 1 – Figure 2. *Change in firing rates of cortical somatosensory neurons. A decrease in firing rate in over 50% of SIo neurons was the criterion for a successful nerve block. Red bars indicate a relative decrease in firing rate for a given electrode, while blue bars indicate an increase. Only electrodes for which the control baseline firing rate was greater than 3 spikes/s are shown. Adapted from Supplementary Figure 6 of "Loss of oral sensation impairs feeding performance and consistency of tongue–jaw coordination" (Laurence-Chasen et al., 2022).*

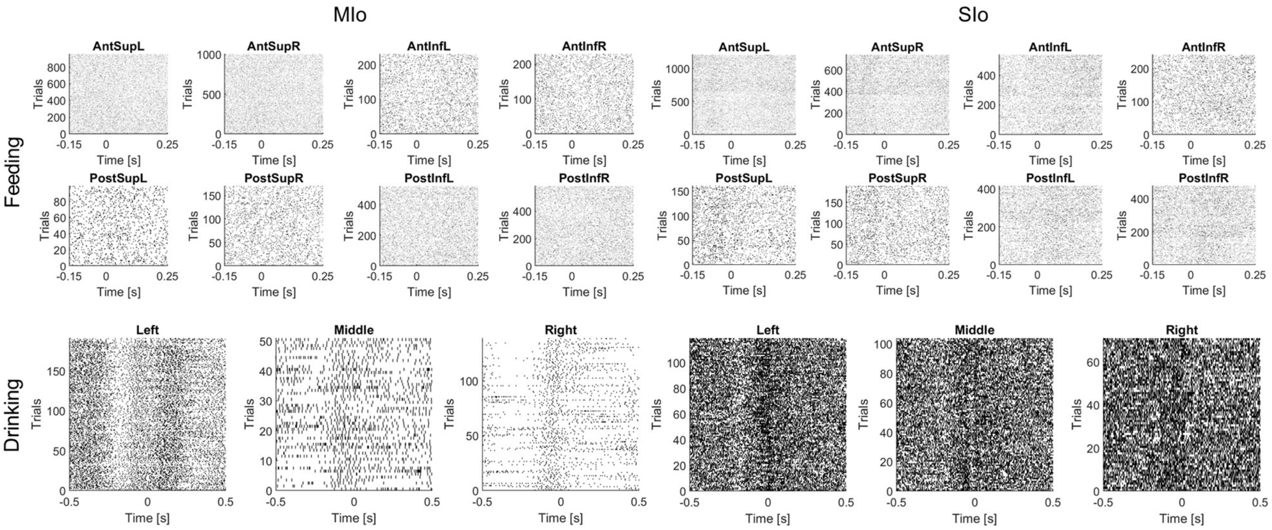

Figure 2 – figure supplement 1. *Directional neural responses across trials. Raster plots corresponding to the same neurons represented in Figure 2. All trials in the respective dataset are included, grouped by direction.*

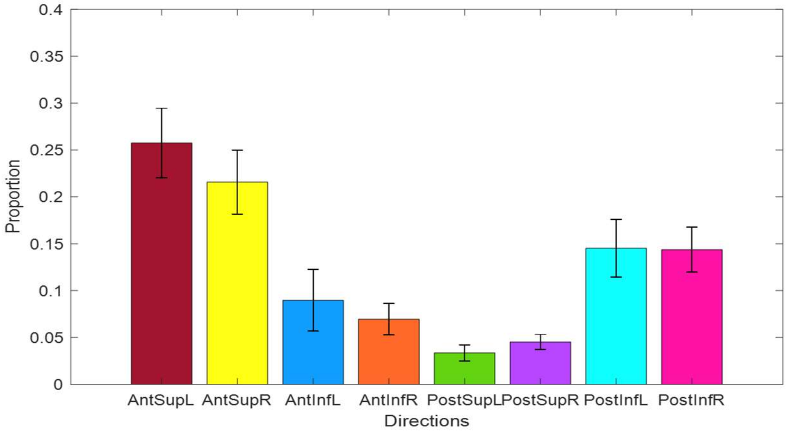

Figure 3 – figure supplement 1*. Proportion of feeding trials in each group of directions. Error bars represent ±1 standard deviation across datasets (n = 4).*

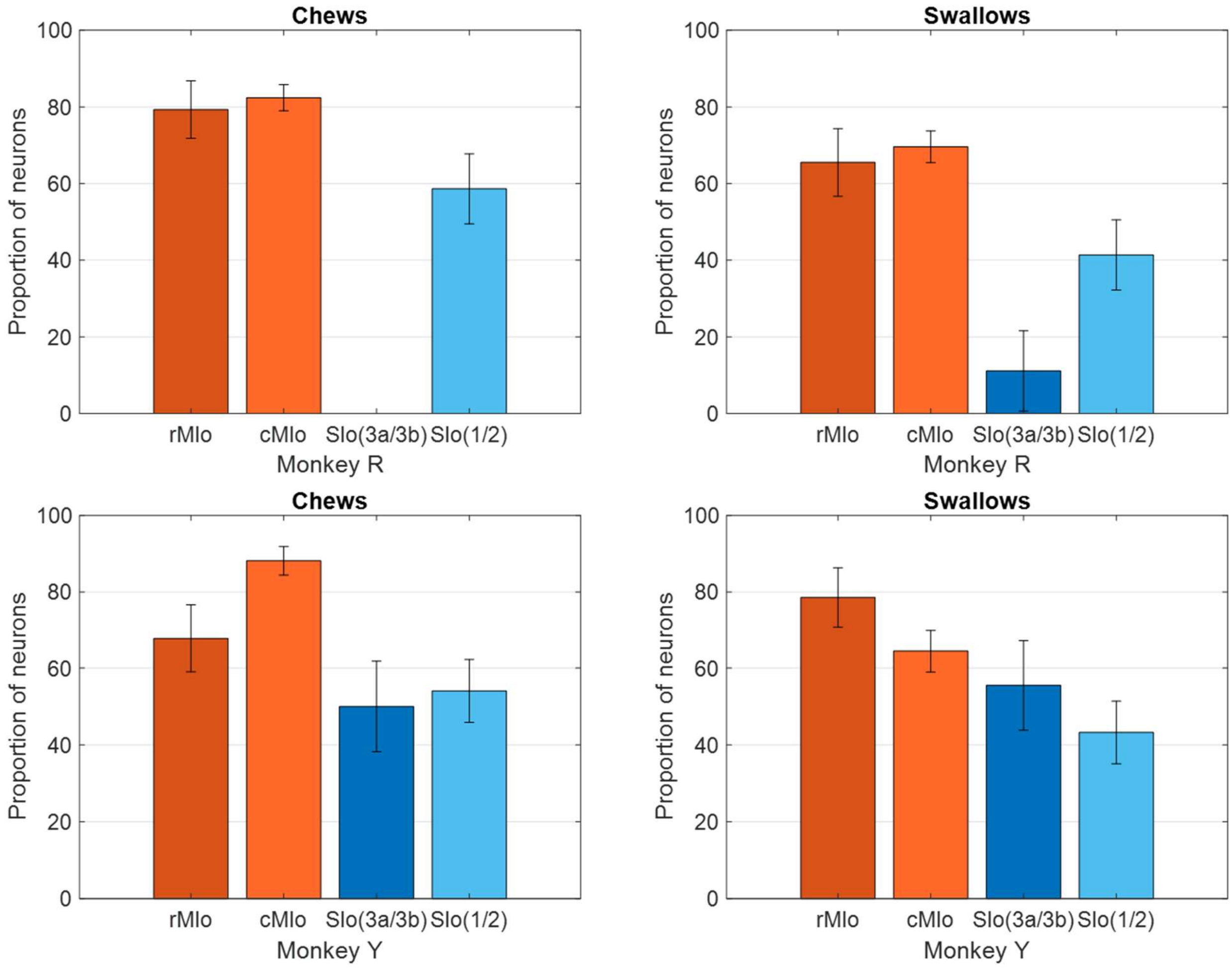

Figure 3 – figure supplement 2. *Proportion of neurons directionally modulated during chews versus swallows in both monkeys. Recordings were taken from four areas of the OSMCx: rMIo - rostral M1, cMIo - caudal M1, SIo(3a/3b) - area 3a/3b, and SIo(1/2) - area 1/2. Error bars represent ±1 SE.*

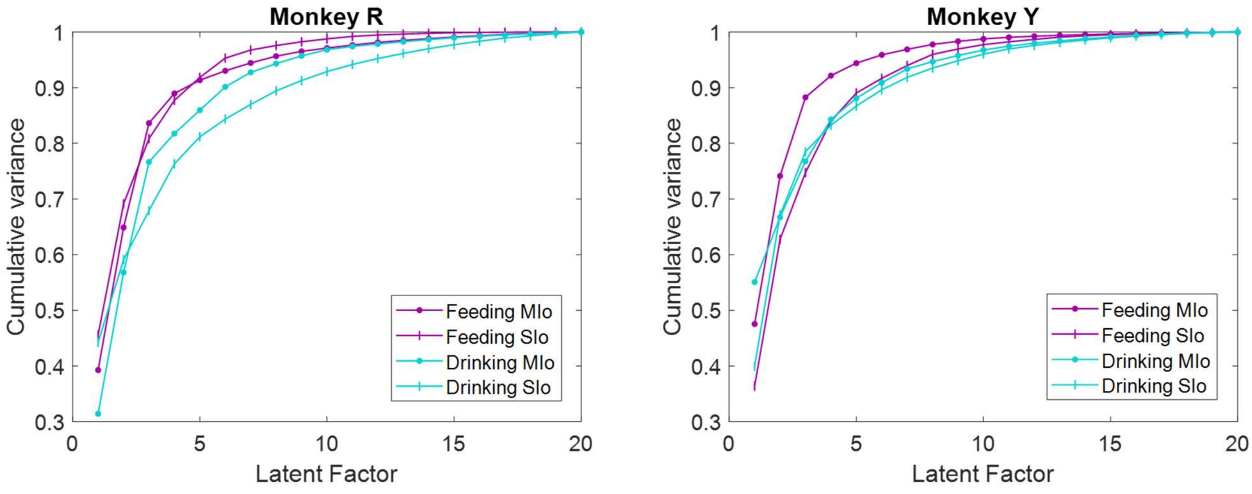

Figure 7 – figure supplement 1. *Comparison of cumulative explained variance between feeding and drinking behaviors with an equal number of neurons (N = 24), for both subjects.*

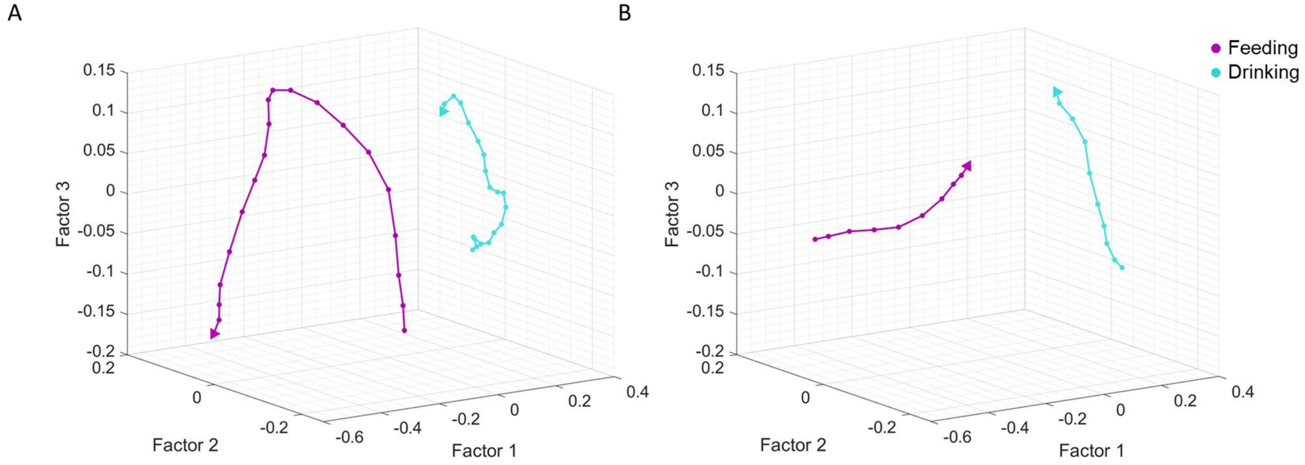

Figure 7 – figure supplement 2. *Comparison between stable MIo (N = 20) neural population trajectories in feeding and drinking behaviors for **(A)** all trials 200 ms around minimum gape and **(B)** a subset of trials (feeding: N = 40, drinking: N = 175) with similar kinematics 100 ms after minimum tongue protrusion. Dots represent each 10 ms time bin and arrow represents the end of the trajectory. Data from monkey Ry.*

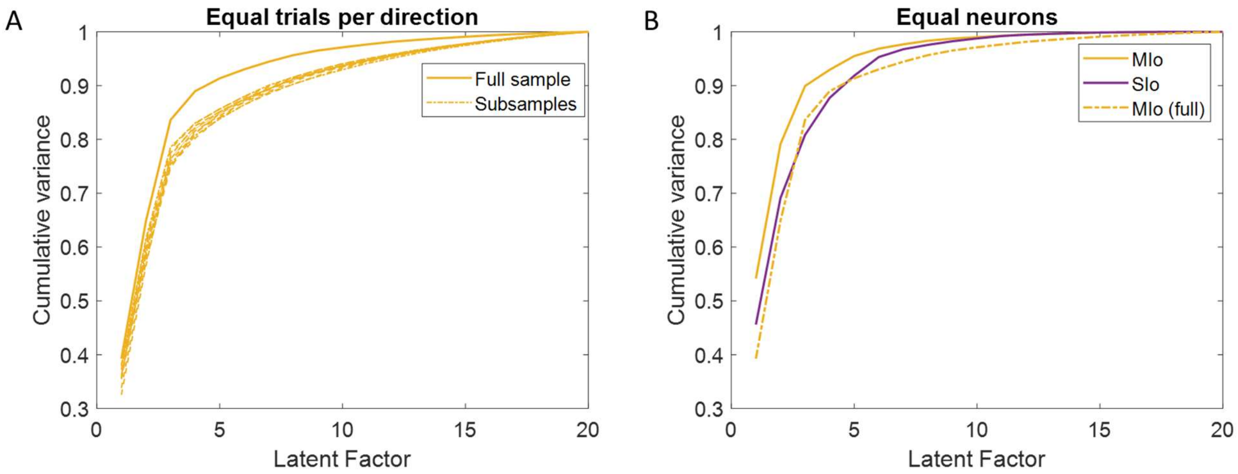

Figure 7 – figure supplement 3. *Effect of subsampling **(A)** equal number of trials per direction (N = 80) from MIo and **(B)** equivalent neuron counts from MIo and SIo populations (N = 24) on the cumulative variance explained by latent factors.*

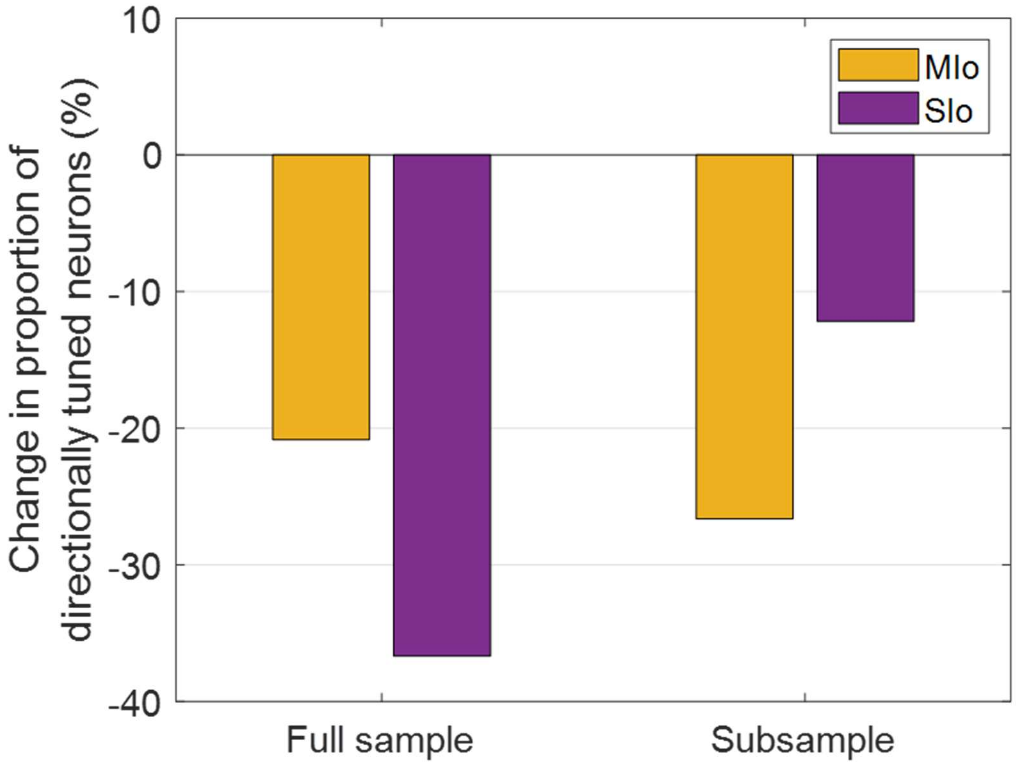

Figure 10 – figure supplement 1. *Effect of subsampling drinking trials with similar kinematic profiles on the change in proportion of directionally tuned neurons in control vs. nerve block conditions for both subjects.*

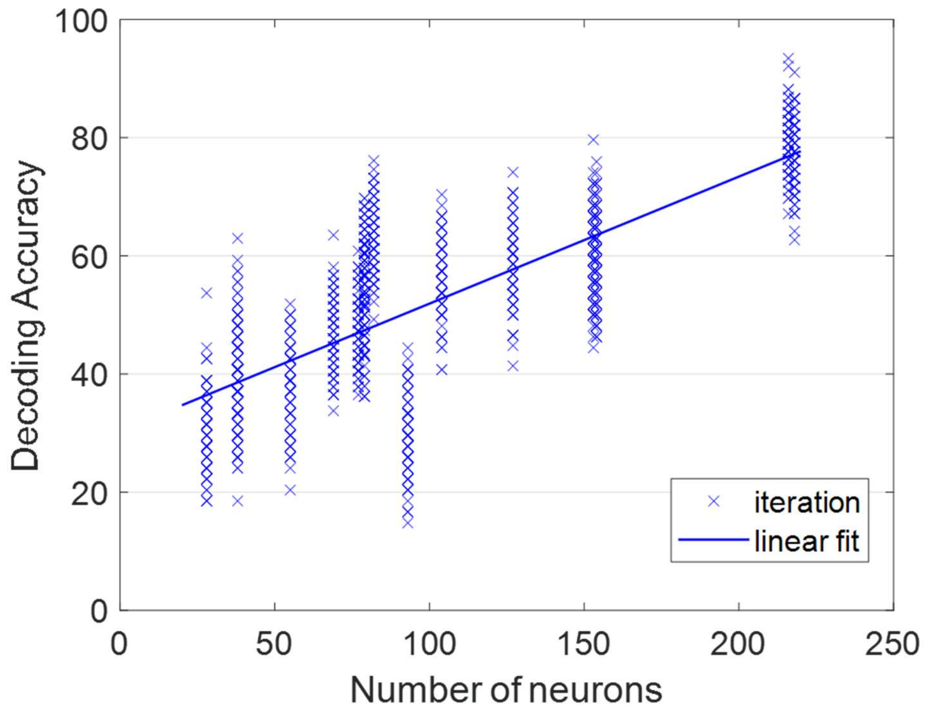

Figure 13 – figure supplement 1. *Correlation between population size and decoding accuracy. Each cross represents one iteration of KNN classification, and the trendline is the linear fit: R^2 = 0.6.*

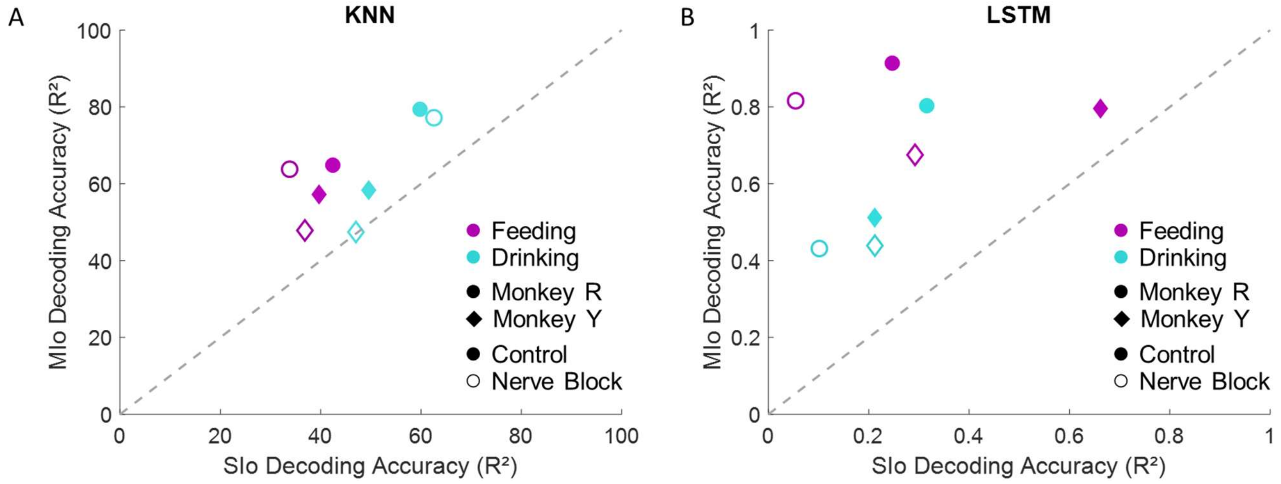

Figure 13 – figure supplement 2. *Decoding accuracies from neuronal populations of various sizes.* ***(A)*** *Comparison between average decoding accuracy of KNN classifier. Chance level is 33.33%.* ***(B)*** *Comparison between average decoding accuracy by LSTM network. Data shown separately for each subject, behavioral task, and condition. The dashed line signifies equal decoding performance for MIo and SIo.*

Control	rMIo	cMIo	SIo(3a/3b)	SIo(1/2)
Ry Feeding	29	125	9	29
Ye Feeding	28	76	18	37
Ry Drinking	31	185	23	54
Ye Drinking	23	104	16	63
**Nerve Block**				
Ry Feeding	27	126	1	27
Ye Feeding	29	64	17	21
Ry Drinking	36	182	26	56
Ye Drinking	22	55	14	55

Supplementary file 1 - Table 1. *Numbers of individual neurons recorded from each array location during each data collection session.*
