# Supplementary material for: 3D directional tuning in the orofacial sensorimotor cortex during natural feeding and drinking
Source: bioRxiv. 2024 Jul 4:2024.07.02.601741. Preprint. [Version 1] doi: 10.1101/2024.07.02.601741 (PMC11244964; doi:10.1101/2024.07.02.601741)
Supplement: Supplementary file 1 [file media-1.docx]

**Supplementary file 1**

**Supplementary figures and tables**

**Supplementary file 1 - Figure** **1****.** Microelectrode array locations.  Squares represent Utah arrays and rectangles represent floating microelectrode arrays. Drawing is to scale and array locations were taken from surgical photographs. Adapted from Supplementary Figure 8 of “Robust cortical encoding of 3D tongue shape during feeding in macaques” (Laurence-Chasen et al., 2023).

**Supplementary file 1 - Figure** **2****.** No effect of sham nerve block on distribution of directions during feeding in Monkey Y. The left half of hemi-violin (black) is control and the right half (red) is sham nerve block. Horizontal black lines represent the mean and horizontal red lines the median. Smaller inset plot is the same variable in the actual nerve block condition (from Fig. 4A), for reference. The sham procedure was identical to the nerve block, except the anesthetic was substituted with saline solution. A two-tailed t-test yielded no significant difference (p > 0.05), compared to the treatment (p < 0.01).

**Supplementary file 1 - Figure** **3****.** No effect of sham nerve block on endpoint variance during drinking in Monkey R. The left half of hemi-violin (black) is control and the right half (red) is sham nerve block. Horizontal black lines represent the mean and horizontal red lines the median. Smaller inset plot is the same variable in the actual nerve block condition for reference. The sham procedure was identical to the nerve block, except the anesthetic was substituted with saline solution. A two-tailed t-test yielded no significant difference (p > 0.1), compared to the treatment (p < 0.001).

**Supplementary file 1 - Figure 4.** Proportion of neurons directionally modulated during chews versus swallows in both monkeys. Recordings were taken from four areas of the OSMCx: rM1 - rostral M1, cM1 - caudal M1, SC(1/2) - area 1/2, and SC(3a/3b) - area 3a/3b. Error bars represent ±1 SE.

**Supplementary file 1 - Figure 5.** Decoding accuracies from neuronal populations of various sizes. Data shown separately for each subject, behavioral task, and condition. The dashed line signifies equal decoding performance for MIo and SIo. Chance level is 33.33%.

**Supplementary file 1 - Figure 6.** Correlation between population size and decoding accuracy. Each cross represents one iteration of KNN classification, and the trendline is the linear fit: R^2^ = 0.6.

 
| **Control** | **rM1** | **cM1** | **SC(1/2)** | **SC(3a/3b)** |
| --- | --- | --- | --- | --- |
| Ry Feeding | 29 | 125 | 9 | 29 |
| Ye Feeding | 28 | 76 | 18 | 37 |
| Ry Drinking | 31 | 185 | 23 | 54 |
| Ye Drinking | 23 | 104 | 16 | 63 |
| **Nerve Block** |  |  |  |  |
| Ry Feeding | 27 | 126 | 1 | 27 |
| Ye Feeding | 29 | 64 | 17 | 21 |
| Ry Drinking | 36 | 182 | 26 | 56 |
| Ye Drinking | 22 | 55 | 14 | 55 |

**Supplementary file 1 - Table** **1****.** Numbers of individual neurons recorded from each array location during each data collection session.
